# CD164 regulates proliferation, progression, and invasion of human glioblastoma cells

**DOI:** 10.18632/oncotarget.26724

**Published:** 2019-03-12

**Authors:** Chung-Ching Wang, Dueng-Yuan Hueng, Ai-Fang Huang, Wei-Liang Chen, Shih-Ming Huang, James Yi-Hsin Chan

**Affiliations:** ^1^ Graduate Institute of Medical Sciences, National Defense Medical Center, Taipei City 114, Taiwan, Republic of China; ^2^ Division of Family Medicine, Department of Family and Community Medicine, Tri-Service General Hospital, and School of Medicine, National Defense Medical Center, Taipei City 114, Taiwan, Republic of China; ^3^ Department of Biochemistry, National Defense Medical Center, Taipei City 114, Taiwan, Republic of China; ^4^ Department of Neurological Surgery, Tri-Service General Hospital, National Defense Medical Center, Taipei City 114, Taiwan, Republic of China; ^5^ Department of Medical Research, Tri-Service General Hospital, National Defense Medical Center, Taipei City 114, Taiwan, Republic of China; ^6^ Department of Microbiology and Immunology, National Defense Medical Center, Taipei City 114, Taiwan, Republic of China; ^7^ Superintendent’s Office, National Defense Medical Center, Taipei City 114, Taiwan, Republic of China

**Keywords:** CD164, human glioma, glioblastoma, autophagy

## Abstract

Grade IV astrocytoma, also known as glioblastoma multiforme (GBM), is the most common and aggressive intracranial glial tumor. GBM is associated with very poor survival and effective treatments have remained elusive so far. Mounting evidence indicates that CD164 contributes to stemness and tumorigenesis in normal cells and is overexpressed in various tumor types, including glioblastoma. Using tissue microarray immunohistochemistry, we show that there is a significant correlation between CD164 expression and glioma type and grade. Depletion of CD164 expression in human glioblastoma cells with siRNA reduced proliferation, migration, and invasiveness. In parallel, immunoblotting showed that downregulation of CD164 expression decreased Akt activation and modified the expression of autophagy markers by upregulating Beclin-1 and LC3B and downregulating p62. These effects were mimicked by inhibition of Akt with MK2206, which suggests that CD164 induces autophagy via Akt/Beclin-1 signaling. We propose that CD164 may serve as a GBM molecular marker and a potential target in therapeutic strategies aimed to improve outcomes for this devastating brain tumor.

## INTRODUCTION

Intracranial tumors are one of the most feared types of cancer. Glioblastoma multiforme (GBM), the most common and most aggressive brain tumor, comprises 15% of all intracranial neoplasms and 60–75% of all astrocytomas [1]. Standard treatment of GBM comprises surgical resection followed by concurrent temozolomide and radiotherapy [2, 3]. However, the overall survival of GBM patients is only 15–18 months [4, 5]. The 2016 World Health Organization (WHO) Classification of Tumors of the Central Nervous System (CNS) was the first publication to list molecular and histology parameters to describe brain tumor entities [6]. Its aim was to simplify clinical and experimental studies to improve survival rates among brain tumor patients. Aided by this information, discovery of new therapeutic molecular targets and individualized treatment approaches to brain tumors are intense areas of research.

Molecular cancer expression signatures are used to predict pathogenicity and survival. PRECOG (PREdiction of Clinical Outcomes from Genomic profiles, available at http://precog.stanford.edu) is a recently stated genomic database that links molecular disease information with survival outcomes [7]. Analysis of PRECOG glioma datasets revealed that the expression of cluster of differentiation 164 (CD164; endolyn), a transmembrane isoform of the mucin-like glycoprotein MGC-24, correlated negatively with survival outcomes in different glioma subsets. This finding triggered our interest in studying the potential contribution of CD164 to GBM biology.

The expression of CD164 characterizes hematopoietic stem cells but is low or negligible in mature peripheral blood neutrophils and erythrocytes [8]. CD164 was shown to regulate the proliferation, adhesion, and differentiation of hematopoietic stem cells [9], and accumulating evidence indicates its potential value as an investigative marker of tumorigenesis and stemness in different cancer types [10–16]. For example, CD164 is highly expressed in colon cancer, where it promotes proliferation and metastasis by regulating signaling through the CXCL12 (SDF-1) receptor CXCR4 [12]. In prostate cancer, CXCL12/CXCR4 signaling induces the expression of CD164 and promotes homing of cancer cells to the bone marrow [13]. In gliomas, knockdown of CD164 inhibited cell proliferation and promoted apoptosis through the PTEN/PI3K/AKT pathway [14]. In non-small cell lung cancer, the tumor suppressor miR-124 targeted CD164 and suppressed tumor proliferation and aggressiveness [15]. Meanwhile, overexpression of CD164 was shown to promote tumorigenesis in normal human lung and ovary epithelial cells [11, 16]. These studies indicated that CD164 may function as a crucial modulator of tumor progression and may be a promising target for cancer treatment. The present study was designed to determine the potential association between CD164 and glioma type and grade, and to investigate the effects and underlying molecular mechanisms of CD164 depletion on the proliferation, migration, and invasion of GBM cells.

## RESULTS

### CD164 is overexpressed in human glioma and correlates with pathological characteristics

**Figure 1:**
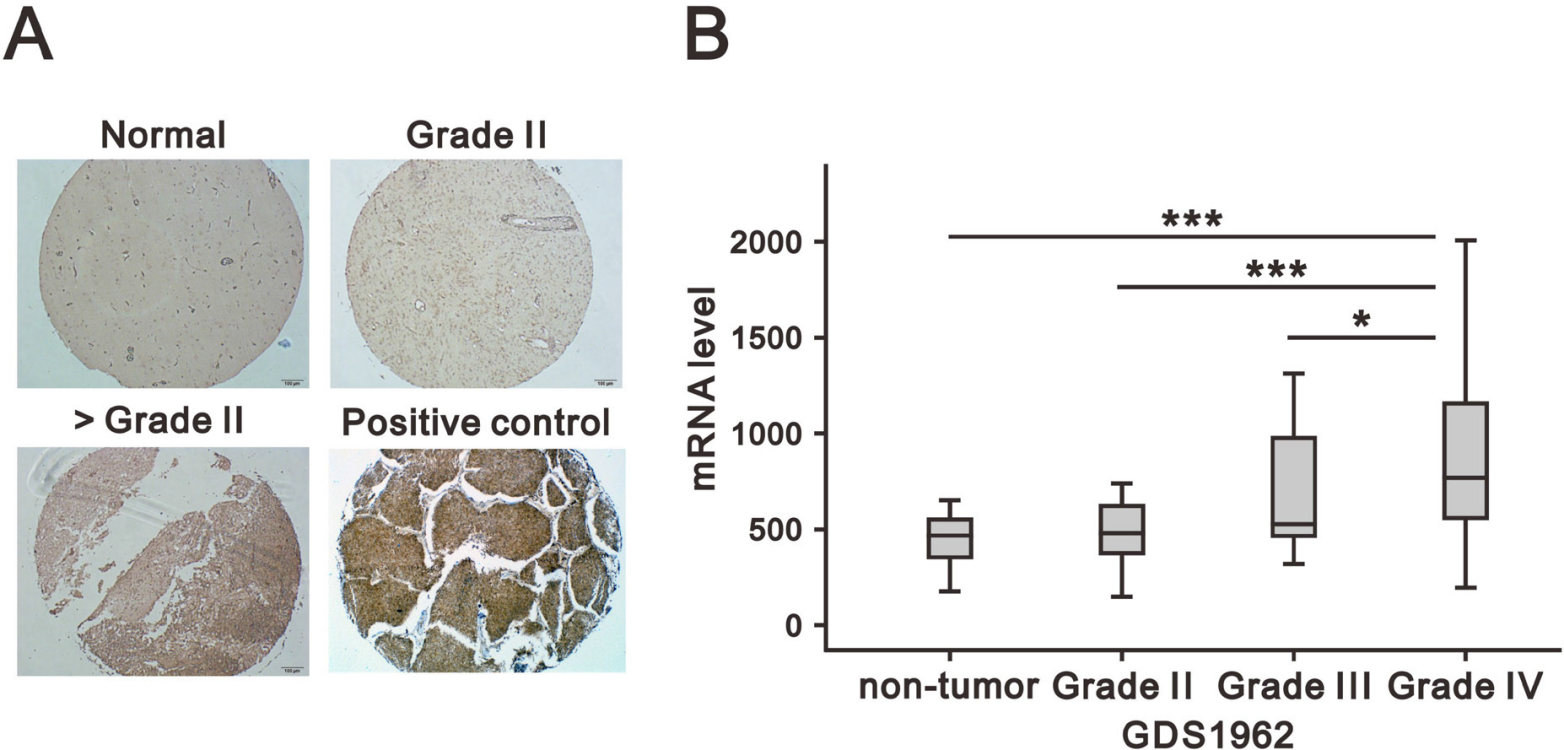
Association of CD164 expression in glioma with clinicopathological parameters (**A**) Representative images of CD164 immunostaining in normal brain and low- and high- grade gliomas. Lung cancer tissue was used as positive control. (**B**) CD164 gene expression analysis in a human glioma microarray dataset (GSD1962) containing 23 non-tumor samples, 76 cases of WHO grade II and III astrocytoma, and 81 cases of GBM (WHO grade IV). mRNA expression was analyzed using ANOVA. ^*^p < 0.05, ^**^p < 0.01, and ^***^p < 0.001 versus non-tumor brain tissues.

To determine potential association of CD164 expression patterns with clinicopathological GBM grade, immunohistochemical staining was performed in a tissue microarray that included normal brain and glioma samples of various histological grades. As shown in Figure 1A, CD164 was highly expressed in the cytoplasm and membrane of glioma cells, although heterogeneous staining patterns were observed across glioma samples. Grade III and IV gliomas presented with significantly higher mean CD164 H-scores than both grade II gliomas and normal brain tissue (Table 1). There was a positive correlation between CD164 H-scores and both tumor type and grade (*p* < 0.001; p for trend < 0.001 for both comparisons). CD164 expression showed no association with either age or sex.

**Table 1:** Correlation between clinical characteristics and CD164 expression in human glioma

Clinical characteristics		Patients (%)	CD164 expression	
			H-score (Mean ± SD)	*p*
Age	>40	54 (54%)	16.11 ± 14.94	
	≤40	46 (46%)	15.46 ± 13.85	0.821
Sex	Male	56 (56%)	17.63 ± 16.81	
	Female	44 (44%)	13.34 ± 9.80	0.135
Tumor cell type	Normal brain tissue	20 (20%)	4.72 ± 3.50	
	Astrocytoma	78 (78%)	18.32 ± 14.89	<0.001
	Oligodendroglioma	2 (2%)	22.18 ± 0.48	*p* for trend = 0.081
Grade^*^	0 (Normal brain tissue)	20 (20%)	4.72 ± 3.50	
	II	32 (32%)	17.30 ± 16.85	<0.001
	Above II	42 (42%)	20.95 ± 12.96	*p* for trend <0.001

^*^Grades II∼III: 15 samples; Grade III: 15 samples; Grades III∼IV: 6 samples; Grade IV: 6 samples. Six unknown-grade samples were excluded. Tumor grading according to WHO histological grading system.

In addition, we analyzed CD164 mRNA expression in human glioma specimens by accessing a Gene Expression Omnibus (GEO) dataset (GDS1962). CD164 mRNA expression was significantly higher in grade IV glioma (*p* < 0.001) than in lower glioma grades (Figure 1B). In conclusion, both tissue microarray immunochemistry and gene expression analyses confirmed a positive relationship between CD164 expression and glioma histological grade.

In addition, we used PRECOG, a public online database, to integrate CD164 gene expression and clinical outcome data [7]. CD164 mRNA expression correlated with worse overall survival in two PRECOG glioma (HR = 2.02, 95% CI 1.56–2.63 [17]; HR = 2.13, 95% CI 1.03–4.42 [18]) (Supplementary Figures 1 and 2) and one astrocytoma (HR = 1.70, 95% CI 1.02–2.81 [19]) datasets (Supplementary Figure 3). The corresponding Kaplan–Meier survival curves are shown as Supplementary Data.

### Depletion of CD164 expression decreases glioblastoma cell proliferation, migration, and invasion

**Figure 2:**
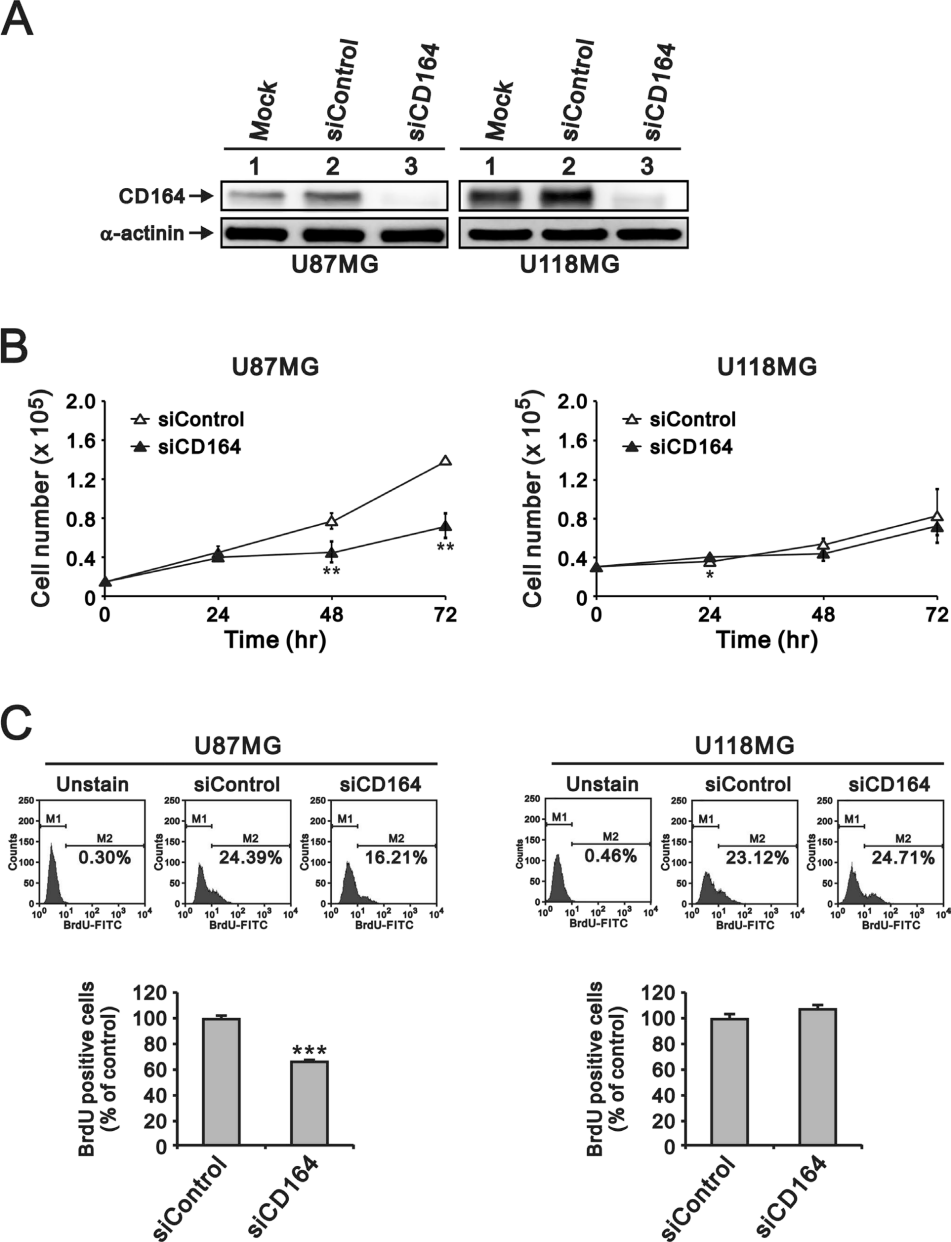
Depletion of CD164 expression decreases proliferation in GBM cells (**A**) Western blot verification of CD164 downregulation after transfection of U87MG and U118MG cells with siCD164. α-actinin was used as loading control. (**B**) Cell proliferation results. Cell numbers were counted at the indicated time points. (**C**) BrdU incorporation assay results. M1, BrdU-negative cells; M2, BrdU-positive cells. Cells not exposed to BrdU were used as blank controls. Results are presented as the mean ± SD of triplicate samples from three independent experiments (^*^p < 0.05, ^**^p < 0.01, ^***^p < 0.001).

To evaluate the potential contribution of CD164 to glioblastoma aggressiveness, human U87MG and U118MG GBM cells were transfected with small interfering (si) RNA targeting the CD164 gene transcript (siCD164). Immunoblotting analyses confirmed that siCD164 transfection resulted in significant downregulation of CD164 expression compared with mock-transfected and non-targeted siRNA transfected cells (siControl) (Figure 2A).

**Figure 3:**
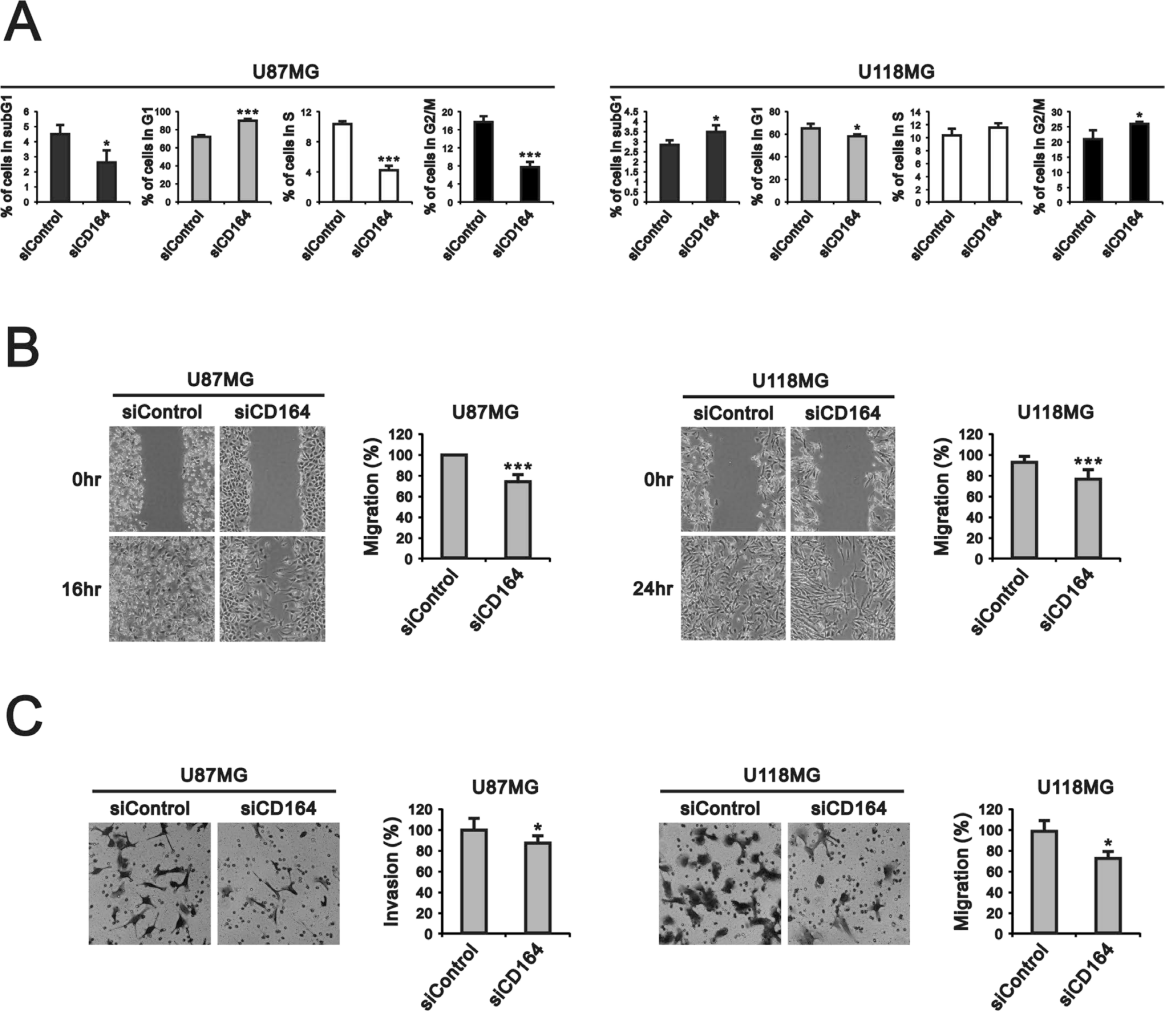
CD164 knockdown alters cell cycle profile and inhibits migration and invasion in GBM cells (**A**) Flow cytometry analysis of cell cycle distribution. (**B**, **C**) Results of migration and invasion assays. Data are presented as the mean ± SD of triplicate samples from three independent experiments (^*^p < 0.05, ^**^p < 0.01, ^***^p < 0.001).

Next, we examined the effects of depleting CD164 expression on the proliferation of U87MG and U118MG cells through cell counting and BrdU assays. CD164 knockdown significantly decreased U87MG cell numbers after 48 h and 72 h, compared to U87MG/siControl cells (Figure 2B). The number of U118MG/siCD164 cells was also lower, compared to U118MG/siControl, after 48 h and 72 h of culture, but this decrease did not show statistical significance (Figure 2B). BrdU incorporation assays showed that silencing of CD164 expression reduced DNA synthesis and cell division, but differences were significant only for U87MG cells (Figure 2C). We also examined cell cycle stages after CD164 silencing. Consistent with the above results, an obvious decrease in the number of cells in S phase was seen in U87MG/siCD164, but not in U118MG/siCD164, cells (Figure 3A).

To evaluate whether silencing of CD164 expression could influence cell migration and invasion, we performed scratch wound and Transwell assays. Results showed that CD164-silenced U87MG and U118MG cells migrated into the cell-free area more slowly than their respective siControl counterparts. Data quantification revealed 100% vs 76.3% area coverage at 16 h for siControl and siCD164 U87MG cells, respectively, and 93.1% vs 76.3% coverage at 24 hr for siControl and siCD164 U118MG cells (Figure 3B). Results from the Transwell invasion assay also indicated impaired invasion capacity after siCD164 silencing in both cell lines (Figure 3C).

### Downregulation of CD164 decreases signaling through the Akt pathway

**Figure 4:**
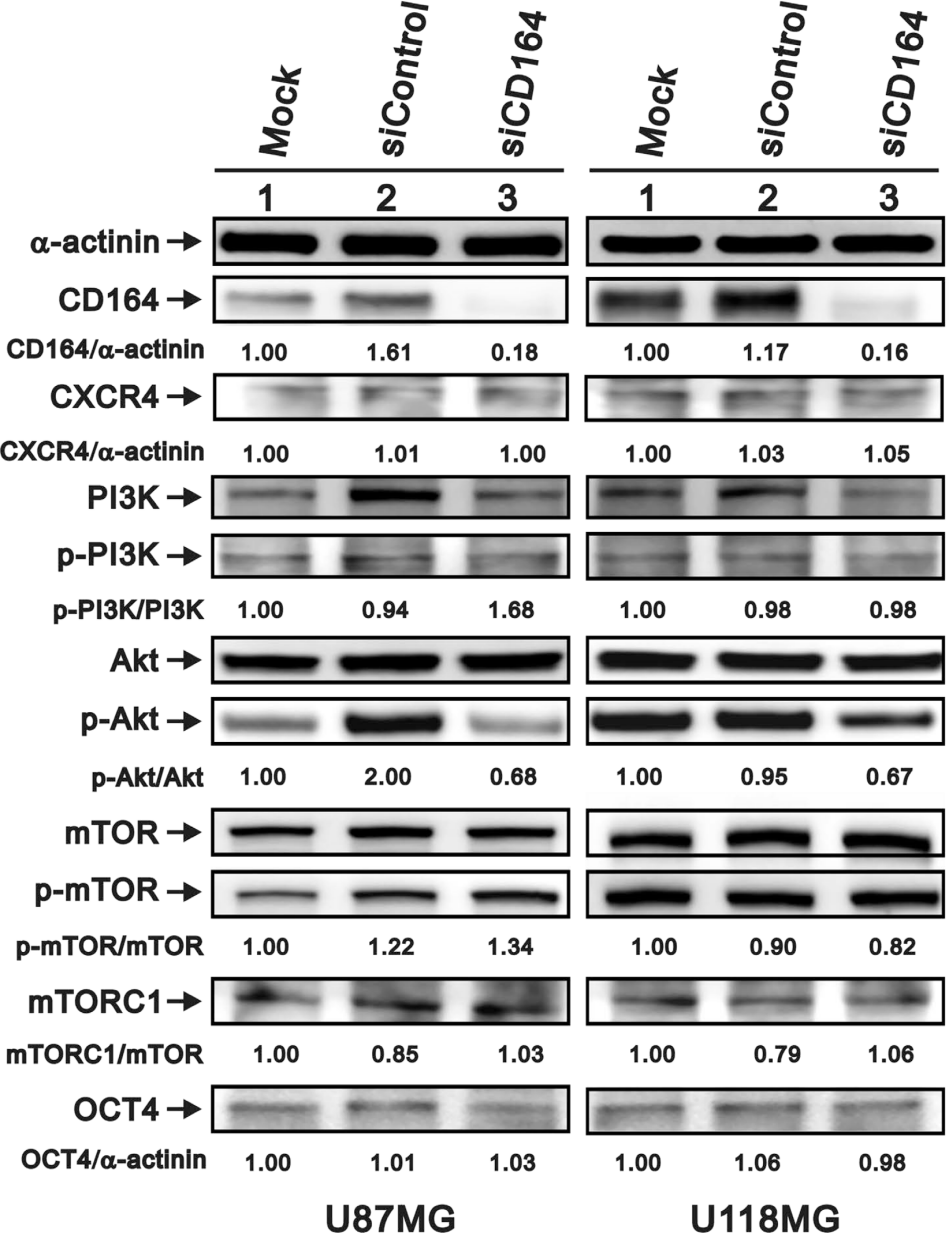
Effects of CD164 knockdown on the CXCR4/PI3K/Akt signaling pathway Immunoblotting analyses were utilized to determine the levels of CXCR4, PI3K (PI3Kp85), total Akt, p-Akt, total mTOR, p-mTOR, mTORC1, and OCT4 in siCD164- and siControl-transfected U87MG and U118MG cells. α-actinin served as loading control. Results are representative of two independent experiments.

Studies have found that CD164 overexpression promotes cancer cell migration by activating the CXCR4/PI3K/AKT/mTOR axis [12, 20–23]. Therefore, we used western blotting to assess the potential modulation of this signaling pathway after downregulation of CD164 in GBM cells. Data showed that CXCR4 and p-PI3K/PI3K expression levels were unchanged in siCD164-transfected U87MG and U118MG cells (Figure 4). In contrast, CD164 silencing reduced the p-Akt/Akt ratio in both cell types. Meanwhile, the ratio of p-mTOR/mTOR was increased in siControl and siCD164 compared with mock in U87MG cells, but decreased in U118MG/siCD164 cells. We next evaluated the expression of mTORC1, one of the two protein complexes that contain mTOR. The ratio of mTORC1/mTOR was decreased in siControl compared with mock and siCD164 in U87MG and U118MG cell lines. Meanwhile, the expression of OCT4, a stem cell marker upregulated by CD164 overexpression in ovarian epithelial cells [16], was unaffected by CD164 silencing in either cell line. Taken together, our data suggests that downregulation of CD164 expression attenuates signaling through the Akt pathway, which correlates with the suppression of proliferation, migration, and invasion observed in GBM cells.

### CD164 downregulation increases autophagy marker expression

**Figure 5:**
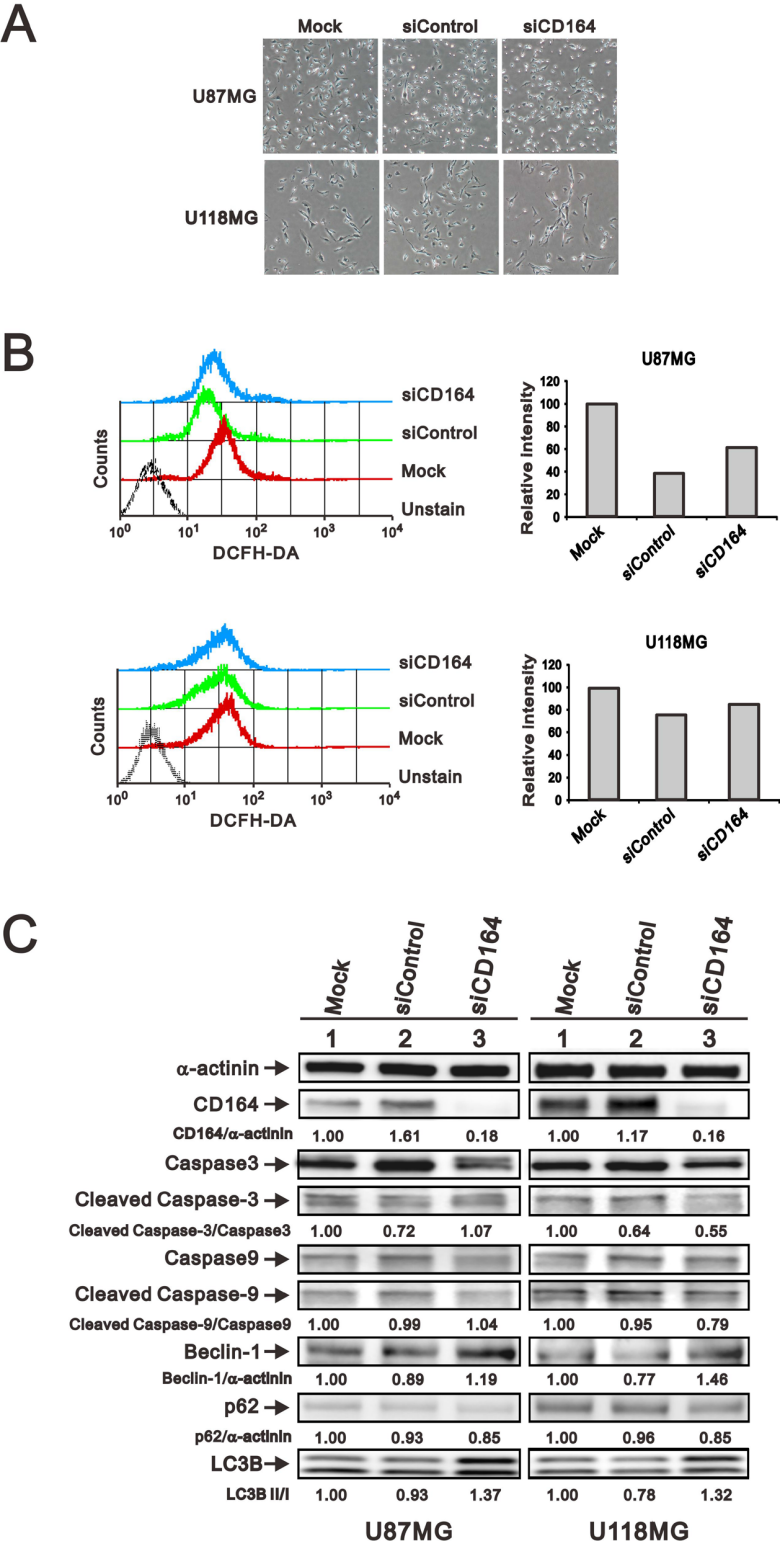
Effects of CD164 depletion on apoptosis, autophagy, and senescence in GBM cells (**A**) Representative images of SA-β gal staining in siCD164- and siControl-transfected U87MG and U118MG cells. (**B**) Intracellular ROS generation in GBM cells was detected by flow cytometry after incubation with DCFH-DA. Cells incubated without DCFH-DA were used as control. (**C**) Immunoblotting analyses of apoptosis markers (total and cleaved caspase 3/9) and autophagy markers (Beclin-1, p62, LC3B, Bcl2, and p-Bcl2) in GBM cells. α-actinin was used as loading control. Results are representative of two independent experiments.

Several processes, among them senescence, oxidative stress, apoptosis, and autophagy can affect cell proliferation and viability. To examine whether downregulation of CD164 in GBM cells had an impact on these events, we examined senescence-associated β-galactosidase (SA-β gal) staining to assess cellular senescence, DCFH-DA fluorescence as a surrogate for reactive oxygen species (ROS) generation associated to oxidative stress, and caspase 3/9 and LC3B expression to assess apoptosis and autophagy respectively. Regardless of CD164 expression status, we observed undetectable SA-β-gal activity (Figure 5A), comparable DCFH-DA fluorescence intensity (Figure 5B), and similar total and active (cleaved) caspase 3/9 levels among GBM cells (Figure 5C). In contrast, the expression of LC3B was higher in siCD164-transfected cells compared to controls (Figure 5C). To further evaluate possible changes in autophagy-related proteins induced by CD164 silencing, the expression of the autophagosome markers Beclin-1 and p62 was examined. Compared with mock and siControl cells, the expression of Beclin-1 was increased, while that of p62 was decreased, in siCD164-transfected cells (Figure 5C). These data suggest that CD164 expression in glioblastoma inhibits autophagy through modulation of LC3B, Beclin-1, and p62 levels.

### Role of Akt in CD164-mediated autophagy inhibition

**Figure 6:**
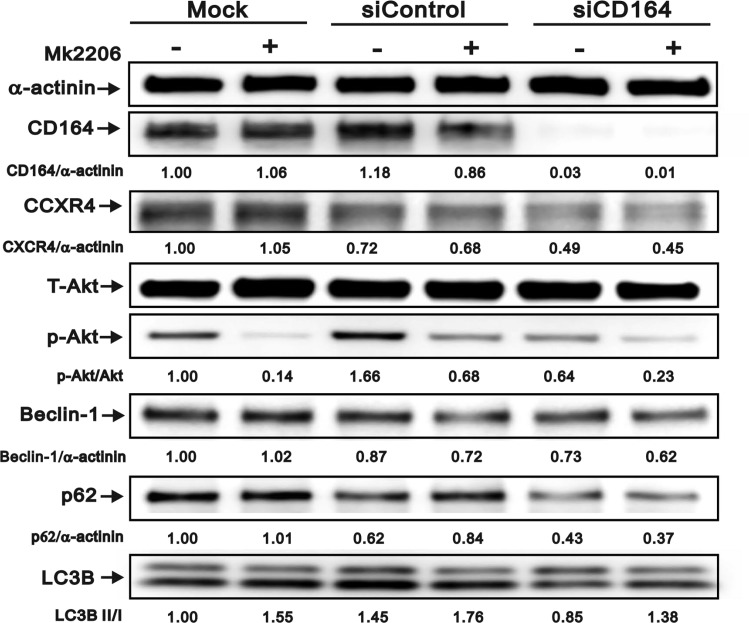
Effect of Akt inhibition on autophagy marker expression in GBM cells Immunoblotting analyses of CD164, CXCR4, total Akt, p-Akt, Beclin-1, p62, and LC3B in GBM cells treated with the Akt inhibitor MK2206. α-actinin served as loading control. Results are representative of two independent experiments.

We further explored the potential effects of Akt inhibition on autophagy protein levels using the Akt inhibitor MK2206 (Figure 6). Treatment of U87MG cells with MK2206 (0.1 µM) for 24 h reduced pAkt/Akt ratio and LC3B in all transfection conditions. Compared with mock and siControl, p62 was decreased in siCD164 cells treated with MK2206. In addition, decreased expression of Beclin-1 and CXCR4 was observed in siControl and siCD164 cells, but not in mock cells, after MK2206 treatment. Collectively, these data suggest that CD164 expression inhibits autophagy through CXCR4/Akt pathway activation and or Beclin-1 regulation.

## DISCUSSION

The expression of the cell-surface sialomucin CD164 is characteristic of human CD34^+^ hematopoietic progenitor cells [9, 24]. However, over the years evidence has shown that CD164 can promote tumorigenesis, invasion, and metastasis in lymphoma [25, 26], prostate cancer [13], colon cancer [12], ovary cancer [16], lung cancer [11, 15] and brain cancer [14, 21]. The present study systematically evaluated CD164 expression in clinical glioma specimens and the effects of CD164 silencing on cultured glioblastoma cells. Tissue microarray analyses showed that CD164 expression positively correlated with glioma type and grade. Further evidence for the adverse impact of CD164 expression on GBM progression was found upon analysis of astrocytoma/glioma PRECOG datasets, which showed a significant, inverse correlation between CD164 levels and survival rates. Further, we showed that knockdown of CD164 expression significantly suppressed the proliferation, migration, and invasion of human U87MG and U118MG GBM cells, and these effects correlated with reduced AKT/mTOR pathway activity and increased autophagy.

A study involving human ovarian surface epithelial cells suggested that CD164 acts as a transcriptional factor that translocates from the cell membrane to the nucleus to induce SDF-1/CXCR4 expression [16]. The same study reported that several stem cell-specific transcriptional factors, such as Nanog, Oct4 and Sox-2, were also activated by CD164 overexpression and this activation increased tumor cell stemness and tumorigenesis. On the other hand, in normal lung epithelial cells overexpression of CD164 also correlated with tumoral transformation by promoting CXCR4 expression and activation of Akt/mTOR signaling [11]. Overproduction of phospholipid and phosphatidylinositol (3, 4, 5) trisphosphate (PIP3) by PI3K and downstream protein kinase Akt has also been noted in a wide range of tumors [27]. Differential activation of SDF-1/CXCR4 signaling, stem cell specific transcriptional factors, PI3K, Akt, and mTOR have been related to glioblastoma progression and aggressiveness. In pediatric gliomas, for instance, activation of the PI3K/Akt/mTOR pathway has been associated with PTEN promoter methylation, which is also found in >80% of secondary adult GBM cases [28]. Therefore, based on the present study as well as current evidence, downregulation of CD164 is predicted to suppress CXCR4-mediated signaling and reduce expression/activation of downstream effectors such as OCT4, Akt, and mTOR.

The reduction in the p-Akt/Akt ratio observed in U87MG and U118MG cells after CD164 knockdown is consistent with the concomitant reduction observed in cell proliferation, migration, and invasion. TP53, PTEN, IDH1, PIK3CA, and EGFR, i.e. the most frequently mutated genes in GBM, are also closely related to the PI3K/Akt pathway [29] and are known to be important regulators of autophagy [30, 31]. Our study suggests that CD164 connects Akt and autophagy and contributes to promotion of GBM growth. However, the function of autophagy in brain cancer and other tumors is still controversial. Studies have reported that inhibition of autophagy by blockade of PI3K and mTOR induced apoptosis, and this effect could be reversed by activation of Akt [32, 33]. Research has also shown that combined suppression of Akt and EGFR with gefitinib and MK2206 induced a switch from autophagy to apoptosis in a mouse glioblastoma model [34]. Altogether, these data imply a central and complex role of Akt in GBM progression, mediated by its impact on autophagy and apoptosis.

Beclin-1 was first identified as a Bcl-2 binding protein that induces autophagy [35]. In addition to Beclin-1, abnormal expression or mutations in Bcl2, mTOR, PI3k, and p53 have been shown to connect autophagy with cancer development [36]. Beclin-1 phosphorylation by Akt positively regulates autophagy and tumorigenesis [37, 38], and studies have found that activation of autophagy by upregulation of LC3B and Beclin-1 decreases viability in GBM cells [39–41]. It was also reported that loss of Beclin-1 results in failure of autophagy and cancer promotion mediated by accumulation of p62 [42]. These results indicate that Akt, Beclin-1, and p62 mediate a complex connection with autophagy in different cells.

We show here the Akt inhibitor MK2206 mimicked the pro-autophagic effects of CD164 silencing in that both induced a decrease in Beclin-1 and p62 and an increase in LC3B. The ratio of LC3B was an indicator of autophagy. However, LC3B itself is degraded by autophagy. An alternative method for detecting the autophagic flux is measuring p62 degradation [43]. In our study, we found that siCD164 or MK2206 treated GBM had mimic change on p62 and LC3B. In addition, the Beclin-1 is important for localization of autophagic protein to a pre-autophagosomal structure and had role between apoptosis and autophagy [44]. The uneven results in our study might be implied the fate of the GBM. Autophagy has been variously proposed to promote or prevent cancer cell growth and invasion, and this may be related to activation or repression of metastatic programs controlled, at least in part and in several tumors including glioma, by the SDF-1/CXCR4 pathway [45–47]. Along these lines, it is tempting to speculate that CD164 expression might regulate autophagy through the CXCR4/Akt/Beclin-1 pathway.

**Figure 7:**
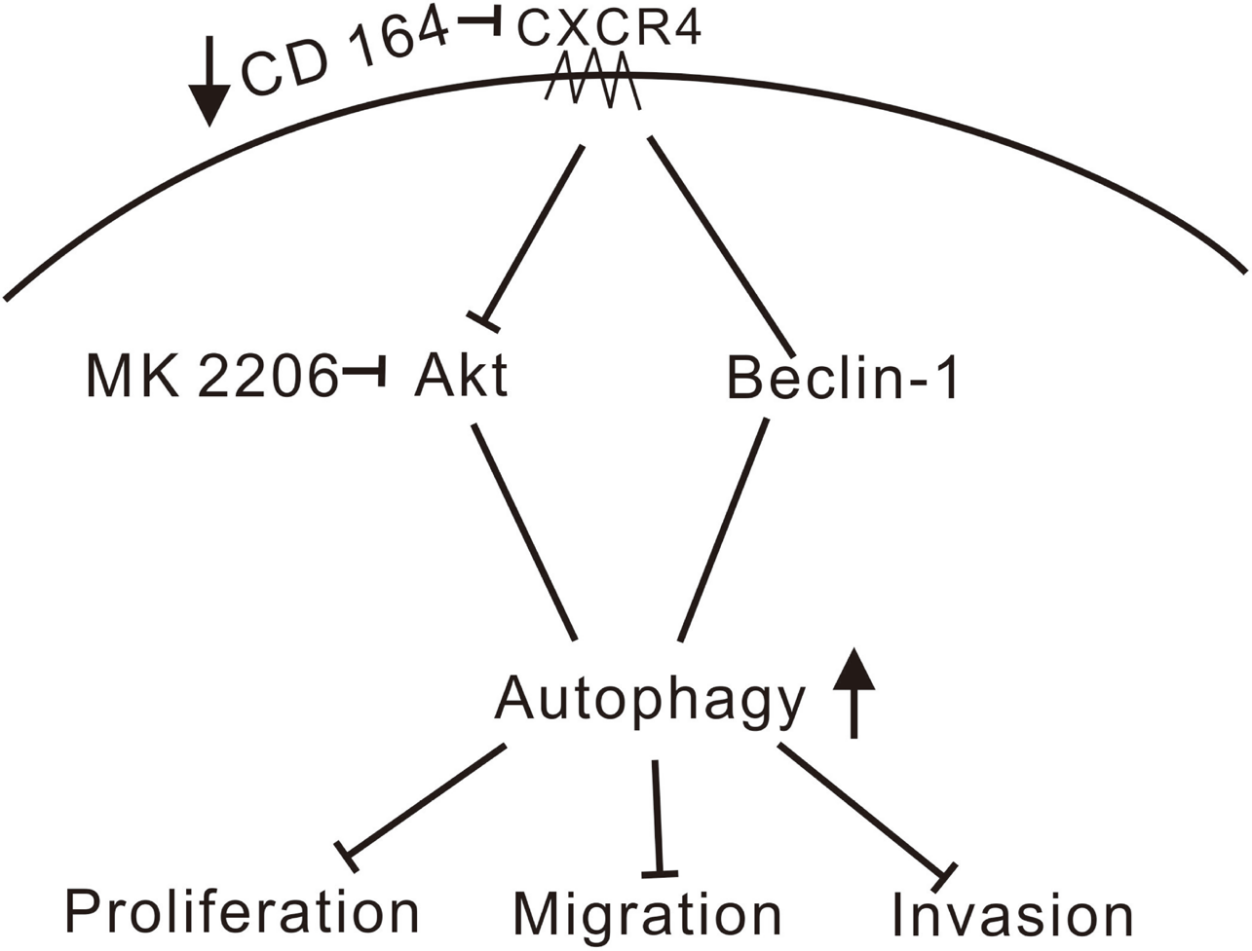
Schematic model of signaling pathways affected by CD164 in GBM We propose that depletion of CD164 leads to suppression of proliferation, migration, and invasion in glioblastoma cells through activation of the CXCR4/Akt/Beclin-1 pathway, resulting in the induction of autophagy. (↑increase, ↓decrease, ⊥inhibition).

Extensive overlap exists between the autophagic and apoptotic machineries, and while both autophagy and apoptosis can be triggered by common upstream signals in response to genotoxic or pharmacological stresses, details of the crosstalk between both processes have not yet been fully elucidated [48, 49]. Interestingly, a previous study found that downregulation of CD164 by short hairpin RNA inhibited cell proliferation and promoted apoptosis in human U87MG glioma cells both *in vitro* and *in vivo* via the PTEN/PI3K/Akt signaling pathway [14]. We found instead that autophagy, rather than apoptosis, was activated by siRNA-mediated CD164 knockdown in U87MG cells, in a process involving the Akt/Beclin-1 signaling pathway (Figure 7).

In conclusion, our study suggests that overexpression of CD164 in glioma cells may contribute to tumor growth through enhanced autophagy. Further experiments are warranted to elucidate the conflicting role of CD164 in SDF-1/CXCR4-dependent signaling in GBM, its impact on autophagy and apoptosis, and the ensuing effects on tumor growth and survival.

## MATERIALS AND METHODS

### Cell culture

Human glioblastoma cell lines U87MG and U118MG were obtained from the American Type Culture Collection (ATCC, Manassas, VA, USA). Cells were maintained in Dulbecco’s modified Eagle’s medium (DMEM) supplemented with 4.5 g/L glucose, 2 mM glutamine, 1% penicillin/streptomycin, and 10% inactivated fetal bovine serum in a humidified atmosphere containing 5% CO_2_ at 37° C. Culture medium changes were performed twice weekly.

### Gene expression Omnibus analysis

The GEO GDS1962 dataset was analyzed in our study. It contained a total of 180 human samples, including 23 non-tumor, 45 grade II astrocytoma, 31 grade III or anaplastic astrocytoma, and 81 grade IV astrocytoma or GBM specimens. CD164 mRNA expression values retrieved from [GDS1962/208653_s_at/CD164] were included in the statistical analysis.

### Tissue microarray immunohistochemistry

One brain tissue microarray was analyzed in our study (GL1002, US Biomax Inc., MD, USA). It included 20 normal brain tissues, 78 astrocytomas, and 2 oligodendrogliomas. For histopathological analysis, microarray sections were deparaffinized by xylene, ethanol, and distilled water. After 15 min incubation in an antigen retrieval solution (Target Retrieval, Dakocytomation, Carpinteria, CA, USA), sections were treated with a monoclonal rabbit antibody against CD164 (Sigma Aldrich Corp., St. Louis, MO, USA) diluted 1:100. This was followed by successive incubation with an HRP-conjugated secondary antibody, 3,3′-diaminobenzidine (DAB), and hematoxylin. CD164 immunoreactivity was quantified using the H-score method, which reflects both staining intensity and percentage of stained cells [50].

### Cell proliferation analyses

Cell proliferation rate was examined in U87MG and U118MG cells seeded in 12-well plates (0.15 × 10^5^ and 0.3 × 10^5^ cells/well, respectively), after 24, 48, 72 hours, the cells were trypsinized and counted using a Coulter counter. In addition, cell proliferation was measured by fluorescence activated cell sorting (FACS) by assessing bromodeoxyuridine (BrdU) incorporation using a FITC-BrdU Flow Kit (BD Biosciences, San Jose, CA, USA) based on manufacturer’s instructions. A FACSCalibur flow cytometer (BD Biosciences, San Jose, CA) was used. Data are expressed by a proliferation ratio, estimated as (N_siCD164_/N_siControl_), where N_siCD164_/N_siControl_ denotes number of activated siCD164/siControl cells at indicated time points.

### Cell cycle assay

Cell cycle distribution was investigated by flow cytometry. After fixation with 70% ice-cold ethanol at 4° C overnight, cells were washed with cold PBS and RNA digested with RNase A (0.5 mg/ml). Cells were then stained with propidium iodide (PI; 5 mg/ml) in PBS/0.5% Triton x-100 for 30 min at 37° C in the dark before analysis.

### Cell migration and invasion assays

For wound migration assays, U87MG (0.5 × 10^5^) and U118MG (1 × 10^5^) cells were incubated in 12-well plates at confluency, and a cell-free linear zone was created using a pipette tip. Cell migration into the clear area was estimated from microscope images taken at different times. We defined 100% migration as total occupation of the cleared zone by migrating cells.

For invasion assays, serum-starved cells were seeded at a density of 2,000 cells/well in the upper chambers of Transwell inserts coated with Matrigel (BD Bioscience, San Jose, CA). The lower chambers were filled with culture medium containing 10% FBS. After 24-hour incubation, cells that traversed into the lower surface of the inserts were stained with crystal violet and counted using a light microscope.

### Western blot analyses

After homogenizing the samples with RIPA lysis buffer containing protease inhibitors, protein concentrations were detected with the Bradford assay. Before electrophoresis on an SDS-agarose gel, an equal amount of cell lysates was added to protein loading dye and boiled for 5–10 min. The separated proteins were blotted electrophoretically onto a polyvinylidene difluoride membrane (Millipore Corp., Bedford, MA, USA). Nonspecific binding was blocked with 5% nonfat milk in Tris-buffered saline for 1 hour at room temperature. The following primary antibodies were then applied overnight at 4° C: alpha-actinin (Santa Cruz Biotechnology, Santa Cruz, CA, USA), CD164 (R&D Systems, Inc. Minneapolis, USA), CXCR4, Akt/p-Akt, mTOR/p-mTOR, OCT4, caspase-3/cleaved caspase-3, caspase-9/cleaved caspase-9, Beclin-1, p62, LC3B (Cell Signaling, Massachusetts, USA), mTORC1 (Proteintech Group, Inc, Rosemont, USA). After washing, HRP-conjugated secondary antibodies diluted in blocking buffer were applied for 1h at room temperature. Immune complexes were detected by enhanced chemiluminescence, and images captured on X-ray film.

### Reactive oxygen species measurement

Intracellular ROS generation was assessed by staining cells with 2′,7′-dichlorofluorescein-diacetate (DCFH-DA; Sigma-Aldrich, St Louis, MO, USA) based on the manufacturer’s protocol. Briefly, cells were incubated with 10 mM DCFH-DA for 30 min at 37° C, harvested, washed twice with PBS, and analyzed by flow cytometry.

### Senescence-associated β-galactosidase staining

Detection of SA-β-gal activity was performed as previously reported [51]. Briefly, glioblastoma cells were fixed in 2% paraformaldehyde and 0.2% glutaraldehyde in PBS for 10–15 min at room temperature, washed in PBS, and stained with X-gal (5-bromo-4-chloro-3-indolyl-β-D-galactopyranoside; Cell Signaling Technology, Inc.) for 8 h. Cell images were captured from randomly selected fields and positive (green) cells were counted.

### Akt inhibition assay

The AKT inhibitor MK-2206 (8-[4-(1-Aminocyclobutyl)phenyl)-9-phenyl-[1,2,4]triazolo[3,4-f][1,6]naphthyridin-3(2H)-one hydrochloride [1:1] was obtained from Cayman chemical (CAS 1032350-13-2). For experiments, cells were seeded at a density of 1.5 × 10^5^ in 6 cm dishes. The next day, MK2206 (diluted from a stock solution formulated in DMSO) was added to cultures at a concentration of 0.1 µM. After 24-hour exposure, the cells were collected and lysis for immunoblotting assays.

### CD164 knockdown

Small interfering RNA (siRNAs) targeting CD164 (siCD164) and non-targeted siRNA (siGENOME SMARTpool, Dharmacon, Lafayette, CO, USA) were used for CD164 knockdown and control, respectively. Briefly, cells were grown in 6 cm dishes and transiently transfected with siRNA at a final concentration of 25 nM using DharmaFECT^™^ transfection reagent based on the manufacturer’s instructions. Mock-transfected cells were exposed to transfection reagent in the absence of siRNAs, and experiments were performed two days after transfection.

### Statistical analysis

Data are presented as mean ± standard deviation or percentage from three separate experiments. Differences between groups were examined using *t*-test and analysis of variance. Two-sided *p* values ≤ 0.05 were considered significant.

## SUPPLEMENTARY MATERIALS FIGURES



## Abbreviations

GBM: glioblastoma multiforme
CNS: central nervous system
PRECOG: PREdiction of Clinical Outcomes from Genomic profiles
CD164: cluster of differentiation 164
CXCL: C-X-C motif chemokine
siRNA: small interfering RNA
PI3K: phosphoinositide 3-kinase
SA-β-gal: senescence associated β-galactosidase

## References

[1] Ellor SV, Pagano-Young TA, Avgeropoulos NG (2014). Glioblastoma: background, standard treatment paradigms, and supportive care considerations. J Law Med Ethics.

[2] Stupp R, Mason WP, van den Bent MJ, Weller M, Fisher B, Taphoorn MJ, Belanger K, Brandes AA, Marosi C, Bogdahn U, Curschmann J, Janzer RC, Ludwin SK (2005). European Organisation for Research and Treatment of Cancer Brain Tumor and Radiotherapy Groups, and National Cancer Institute of Canada Clinical Trials Group. Radiotherapy plus concomitant and adjuvant temozolomide for glioblastoma. N Engl J Med.

[3] Venur VA, Peereboom DM, Ahluwalia MS (2015). Current medical treatment of glioblastoma. Cancer Treat Res.

[4] Stupp R, Hegi ME, Mason WP, van den Bent MJ, Taphoorn MJ, Janzer RC, Ludwin SK, Allgeier A, Fisher B, Belanger K, Hau P, Brandes AA, Gijtenbeek J (2009). European Organisation for Research and Treatment of Cancer Brain Tumour and Radiation Oncology Groups, and National Cancer Institute of Canada Clinical Trials Group. Effects of radiotherapy with concomitant and adjuvant temozolomide versus radiotherapy alone on survival in glioblastoma in a randomised phase III study: 5-year analysis of the EORTC-NCIC trial. Lancet Oncol.

[5] Clarke JL, Iwamoto FM, Sul J, Panageas K, Lassman AB, DeAngelis LM, Hormigo A, Nolan CP, Gavrilovic I, Karimi S, Abrey LE (2009). Randomized phase II trial of chemoradiotherapy followed by either dose-dense or metronomic temozolomide for newly diagnosed glioblastoma. J Clin Oncol.

[6] Louis DN, Perry A, Reifenberger G, von Deimling A, Figarella-Branger D, Cavenee WK, Ohgaki H, Wiestler OD, Kleihues P, Ellison DW (2016). The 2016 World Health Organization Classification of Tumors of the Central Nervous System: a summary. Acta Neuropathol.

[7] Gentles AJ, Newman AM, Liu CL, Bratman SV, Feng W, Kim D, Nair VS, Xu Y, Khuong A, Hoang CD, Diehn M, West RB, Plevritis SK, Alizadeh AA (2015). The prognostic landscape of genes and infiltrating immune cells across human cancers. Nat Med.

[8] Watt SM, Butler LH, Tavian M, Bühring HJ, Rappold I, Simmons PJ, Zannettino AC, Buck D, Fuchs A, Doyonnas R, Chan JY, Levesque JP, Peault B, Roxanis I (2000). Functionally defined CD164 epitopes are expressed on CD34(+) cells throughout ontogeny but display distinct distribution patterns in adult hematopoietic and nonhematopoietic tissues. Blood.

[9] Doyonnas R, Yi-Hsin Chan J, Butler LH, Rappold I, Lee-Prudhoe JE, Zannettino AC, Simmons PJ, Bühring HJ, Levesque JP, Watt SM (2000). CD164 monoclonal antibodies that block hemopoietic progenitor cell adhesion and proliferation interact with the first mucin domain of the CD164 receptor. J Immunol.

[10] Benoit BM, Jariwala N, O’Connor G, Oetjen LK, Whelan TM, Werth A, Troxel AB, Sicard H, Zhu L, Miller C, Takeshita J, McVicar DW, Kim BS (2017). CD164 identifies CD4+ T cells highly expressing genes associated with malignancy in Sézary syndrome: the Sézary signature genes, FCRL3, Tox, and miR-214. Arch Dermatol Res.

[11] Chen WL, Huang AF, Huang SM, Ho CL, Chang YL, Chan JY (2016). CD164 promotes lung tumor-initiating cells with stem cell activity and determines tumor growth and drug resistance via Akt/mTOR signaling. Oncotarget.

[12] Tang J, Zhang L, She X, Zhou G, Yu F, Xiang J, Li G (2012). Inhibiting CD164 expression in colon cancer cell line HCT116 leads to reduced cancer cell proliferation, mobility, and metastasis *in vitro* and *in vivo*. Cancer Invest.

[13] Havens AM, Jung Y, Sun YX, Wang J, Shah RB, Bühring HJ, Pienta KJ, Taichman RS (2006). The role of sialomucin CD164 (MGC-24v or endolyn) in prostate cancer metastasis. BMC Cancer.

[14] Tu M, Cai L, Zheng W, Su Z, Chen Y, Qi S (2017). CD164 regulates proliferation and apoptosis by targeting PTEN in human glioma. Mol Med Rep.

[15] Lin J, Xu K, Wei J, Heimberger AB, Roth JA, Ji L (2016). MicroRNA-124 suppresses tumor cell proliferation and invasion by targeting CD164 signaling pathway in non-small cell lung cancer. J Gene Ther.

[16] Huang AF, Chen MW, Huang SM, Kao CL, Lai HC, Chan JY (2013). CD164 regulates the tumorigenesis of ovarian surface epithelial cells through the SDF-1α/CXCR4 axis. Mol Cancer.

[17] Nutt CL, Mani DR, Betensky RA, Tamayo P, Cairncross JG, Ladd C, Pohl U, Hartmann C, McLaughlin ME, Batchelor TT, Black PM, von Deimling A, Pomeroy SL (2003). Gene expression-based classification of malignant gliomas correlates better with survival than histological classification. Cancer Res.

[18] Li A, Walling J, Ahn S, Kotliarov Y, Su Q, Quezado M, Oberholtzer JC, Park J, Zenklusen JC, Fine HA (2009). Unsupervised analysis of transcriptomic profiles reveals six glioma subtypes. Cancer Res.

[19] Petalidis LP, Oulas A, Backlund M, Wayland MT, Liu L, Plant K, Happerfield L, Freeman TC, Poirazi P, Collins VP (2008). Improved grading and survival prediction of human astrocytic brain tumors by artificial neural network analysis of gene expression microarray data. Mol Cancer Ther.

[20] Forde S, Tye BJ, Newey SE, Roubelakis M, Smythe J, McGuckin CP, Pettengell R, Watt SM (2007). Endolyn (CD164) modulates the CXCL12-mediated migration of umbilical cord blood CD133+ cells. Blood.

[21] Shi JA, Lu DL, Huang X, Tan W (2014). miR-219 inhibits the proliferation, migration and invasion of medulloblastoma cells by targeting CD164. Int J Mol Med.

[22] Khan KH, Yap TA, Yan L, Cunningham D (2013). Targeting the PI3K-AKT-mTOR signaling network in cancer. Chin J Cancer.

[23] Ping YF, Yao XH, Jiang JY, Zhao LT, Yu SC, Jiang T, Lin MC, Chen JH, Wang B, Zhang R, Cui YH, Qian C, Wang J, Bian XW (2011). The chemokine CXCL12 and its receptor CXCR4 promote glioma stem cell-mediated VEGF production and tumour angiogenesis via PI3K/AKT signalling. J Pathol.

[24] Zannettino AC, Bühring HJ, Niutta S, Watt SM, Benton MA, Simmons PJ (1998). The sialomucin CD164 (MGC-24v) is an adhesive glycoprotein expressed by human hematopoietic progenitors and bone marrow stromal cells that serves as a potent negative regulator of hematopoiesis. Blood.

[25] Guenova E, Ignatova D, Chang YT, Contassot E, Mehra T, Saulite I, Navarini AA, Mitev V, Dummer R, Kazakov DV, French LE, Hoetzenecker W, Cozzio A (2016). Expression of CD164 on Malignant T cells in Sézary Syndrome. Acta Derm Venereol.

[26] Wysocka M, Kossenkov AV, Benoit BM, Troxel AB, Singer E, Schaffer A, Kim B, Dentchev T, Nagata S, Ise T, Showe LC, Rook AH (2014). CD164 and FCRL3 are highly expressed on CD4+CD26- T cells in Sézary syndrome patients. J Invest Dermatol.

[27] Vivanco I, Sawyers CL (2002). The phosphatidylinositol 3-Kinase AKT pathway in human cancer. Nat Rev Cancer.

[28] Mueller S, Phillips J, Onar-Thomas A, Romero E, Zheng S, Wiencke JK, McBride SM, Cowdrey C, Prados MD, Weiss WA, Berger MS, Gupta N, Haas-Kogan DA (2012). PTEN promoter methylation and activation of the PI3K/Akt/mTOR pathway in pediatric gliomas and influence on clinical outcome. Neuro-oncol.

[29] Bleeker FE, Lamba S, Zanon C, Molenaar RJ, Hulsebos TJ, Troost D, van Tilborg AA, Vandertop WP, Leenstra S, van Noorden CJ, Bardelli A (2014). Mutational profiling of kinases in glioblastoma. BMC Cancer.

[30] Kaza N, Kohli L, Roth KA (2012). Autophagy in brain tumors: a new target for therapeutic intervention. Brain Pathol.

[31] Verhaak RG, Hoadley KA, Purdom E, Wang V, Qi Y, Wilkerson MD, Miller CR, Ding L, Golub T, Mesirov JP, Alexe G, Lawrence M, O’Kelly M (2010). Cancer Genome Atlas Research Network. Integrated genomic analysis identifies clinically relevant subtypes of glioblastoma characterized by abnormalities in PDGFRA, IDH1, EGFR, and NF1. Cancer Cell.

[32] Fan QW, Weiss WA (2011). Autophagy and Akt promote survival in glioma. Autophagy.

[33] Fan QW, Cheng C, Hackett C, Feldman M, Houseman BT, Nicolaides T, Haas-Kogan D, James CD, Oakes SA, Debnath J, Shokat KM, Weiss WA (2010). Akt and autophagy cooperate to promote survival of drug-resistant glioma. Sci Signal.

[34] Cheng Y, Zhang Y, Zhang L, Ren X, Huber-Keener KJ, Liu X, Zhou L, Liao J, Keihack H, Yan L, Rubin E, Yang JM (2012). MK-2206, a novel allosteric inhibitor of Akt, synergizes with gefitinib against malignant glioma via modulating both autophagy and apoptosis. Mol Cancer Ther.

[35] Furuya N, Yu J, Byfield M, Pattingre S, Levine B (2005). The evolutionarily conserved domain of Beclin 1 is required for Vps34 binding, autophagy and tumor suppressor function. Autophagy.

[36] Kung CP, Budina A, Balaburski G, Bergenstock MK, Murphy M (2011). Autophagy in tumor suppression and cancer therapy. Crit Rev Eukaryot Gene Expr.

[37] Wang RC, Wei Y, An Z, Zou Z, Xiao G, Bhagat G, White M, Reichelt J, Levine B (2012). Akt-mediated regulation of autophagy and tumorigenesis through Beclin 1 phosphorylation. Science.

[38] Sun PH, Zhu LM, Qiao MM, Zhang YP, Jiang SH, Wu YL, Tu SP (2011). The XAF1 tumor suppressor induces autophagic cell death via upregulation of Beclin-1 and inhibition of Akt pathway. Cancer Lett.

[39] Hou W, Song L, Zhao Y, Liu Q, Zhang S (2017). Inhibition of Beclin-1-Mediated Autophagy by MicroRNA-17-5p Enhanced the Radiosensitivity of Glioma Cells. Oncol Res.

[40] Zhong JT, Xu Y, Yi HW, Su J, Yu HM, Xiang XY, Li XN, Zhang ZC, Sun LK (2012). The BH3 mimetic S1 induces autophagy through ER stress and disruption of Bcl-2/Beclin 1 interaction in human glioma U251 cells. Cancer Lett.

[41] Graf MR, Jia W, Johnson RS, Dent P, Mitchell C, Loria RM (2009). Autophagy and the functional roles of Atg5 and beclin-1 in the anti-tumor effects of 3beta androstene 17alpha diol neuro-steroid on malignant glioma cells. J Steroid Biochem Mol Biol.

[42] Mathew R, Karp CM, Beaudoin B, Vuong N, Chen G, Chen HY, Bray K, Reddy A, Bhanot G, Gelinas C, Dipaola RS, Karantza-Wadsworth V, White E (2009). Autophagy suppresses tumorigenesis through elimination of p62. Cell.

[43] Mizushima N, Yoshimori T (2007). How to interpret LC3 immunoblotting. Autophagy.

[44] Kang R, Zeh HJ, Lotze MT, Tang D (2011). The Beclin 1 network regulates autophagy and apoptosis. Cell Death Differ.

[45] Yadav VN, Zamler D, Baker GJ, Kadiyala P, Erdreich-Epstein A, DeCarvalho AC, Mikkelsen T, Castro MG, Lowenstein PR (2016). CXCR4 increases in-vivo glioma perivascular invasion, and reduces radiation induced apoptosis: A genetic knockdown study. Oncotarget.

[46] Coly PM, Perzo N, Le Joncour V, Lecointre C, Schouft MT, Desrues L, Tonon MC, Wurtz O, Gandolfo P, Castel H, Morin F (2016). Chemotactic G protein-coupled receptors control cell migration by repressing autophagosome biogenesis. Autophagy.

[47] Zeng Y, Wang X, Yin B, Xia G, Shen Z, Gu W, Wu M (2017). Role of the stromal cell derived factor-1/CXC chemokine receptor 4 axis in the invasion and metastasis of lung cancer and mechanism. J Thorac Dis.

[48] Eisenberg-Lerner A, Bialik S, Simon HU, Kimchi A (2009). Life and death partners: apoptosis, autophagy and the cross-talk between them. Cell Death Differ.

[49] Sui X, Kong N, Ye L, Han W, Zhou J, Zhang Q, He C, Pan H (2014). p38 and JNK MAPK pathways control the balance of apoptosis and autophagy in response to chemotherapeutic agents. Cancer Lett.

[50] Detre S, Saclani Jotti G, Dowsett M (1995). A “quickscore” method for immunohistochemical semiquantitation: validation for oestrogen receptor in breast carcinomas. J Clin Pathol.

[51] Dimri GP, Lee X, Basile G, Acosta M, Scott G, Roskelley C, Medrano EE, Linskens M, Rubelj I, Pereira-Smith O (1995). A biomarker that identifies senescent human cells in culture and in aging skin *in vivo*. Proc Natl Acad Sci USA.

