# Supplementary material for: CD164 regulates proliferation, progression, and invasion of human glioblastoma cells
Source: Oncotarget. 2019 Mar 12;10(21):2041–54. doi: 10.18632/oncotarget.26724 (PMC6459350; doi:10.18632/oncotarget.26724)
Supplement: Supplementary file 1 [file oncotarget-10-2041-s001.pdf]

CD164 regulates proliferation, progression, and invasion of human glioblastoma cells

SUPPLEMENTARY MATERIALS

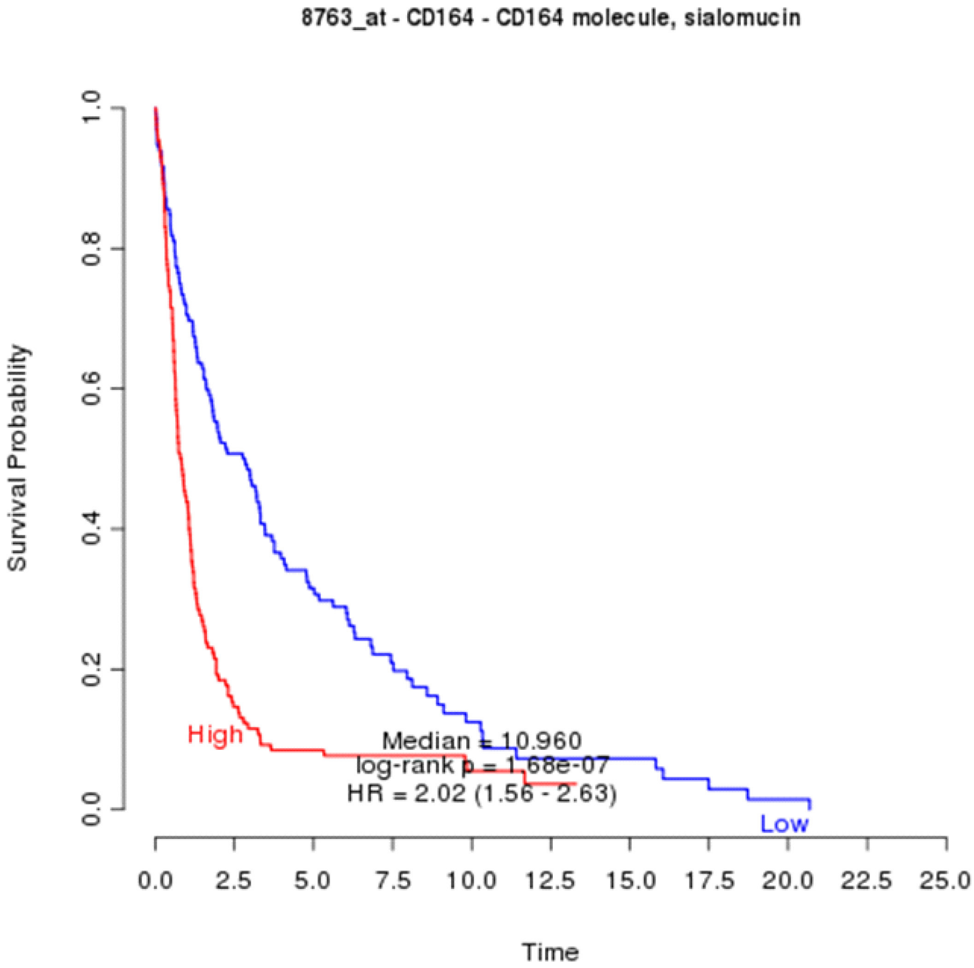

Supplementary Figure 1: KM Plot(s) for gene CD164 in Brain cancer Glioma. PubMed: Gravendeel *et al*. (opens in new browser window/tab). Accession: GSE16011 (opens in new browser window/tab). No. patients (OS/DSS): 255

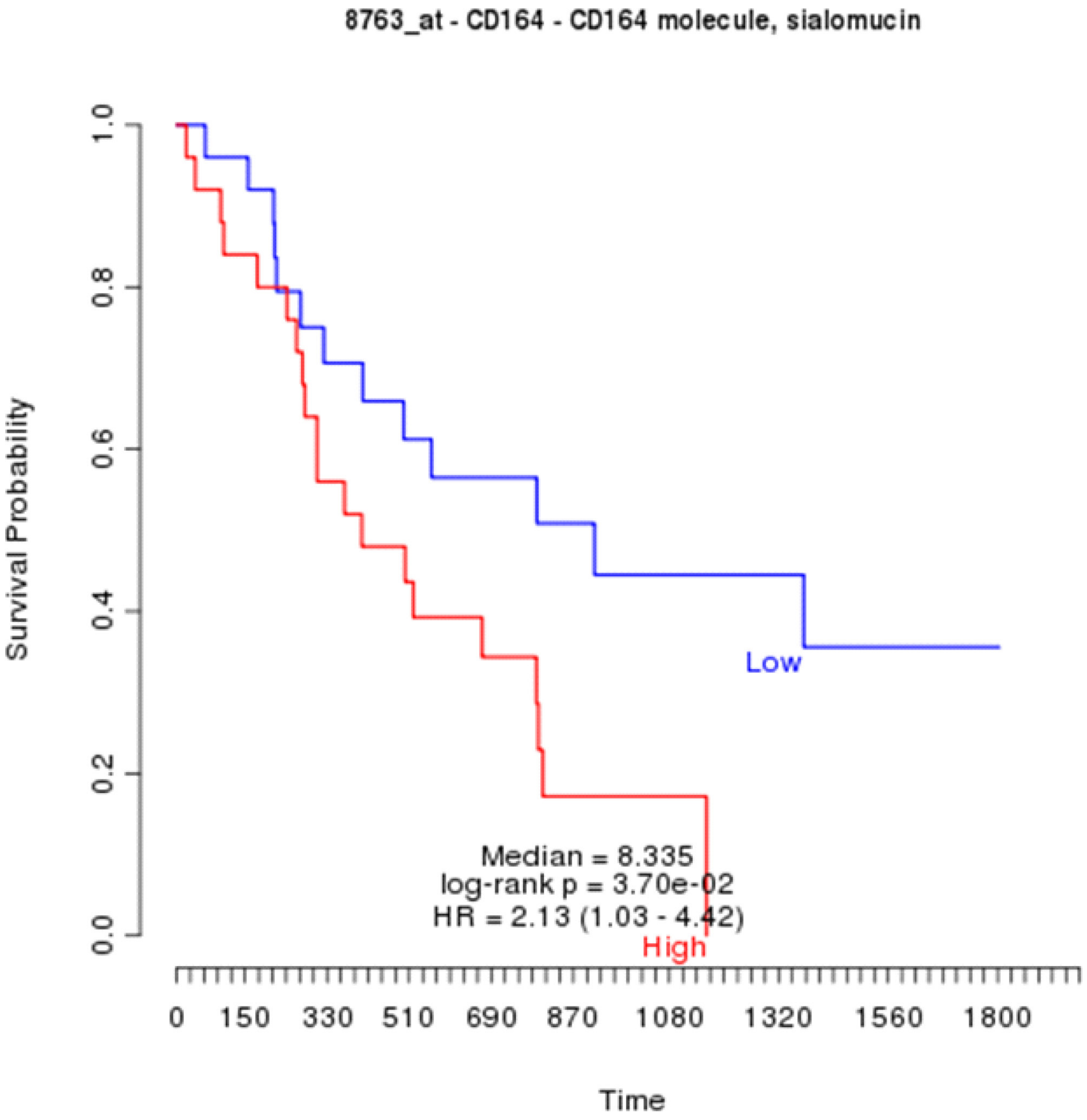

Supplementary Figure 2: KM Plot(s) for gene CD164 in Brain cancer Glioma. PubMed: Nutt *et al*. (opens in new browser window/tab). Accession: Nutt_Glioma. No. patients (OS/DSS): 50.

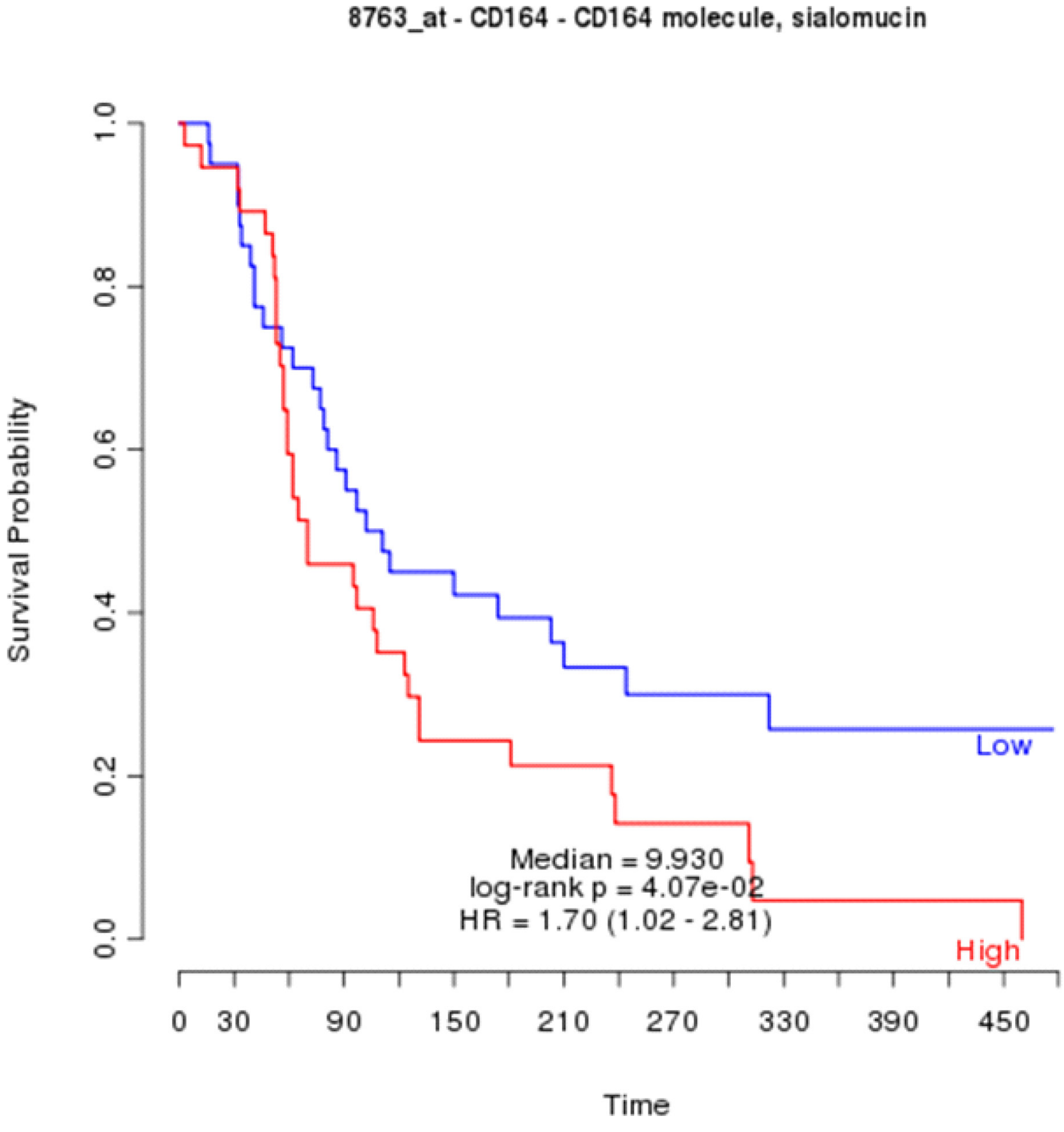

Supplementary Figure 3: KM Plot(s) for gene CD164 in Brain cancer Astrocytoma. PubMed: Phillips *et al*. (opens in new browser window/tab). Accession: GSE4271 (opens in new browser window/tab). No. patients (OS/DSS): 77.
